# Adaptive Carbon Allocation by Plants Enhances the Terrestrial Carbon Sink

**DOI:** 10.1038/s41598-017-03574-3

**Published:** 2017-06-13

**Authors:** Jiangzhou Xia, Wenping Yuan, Ying-Ping Wang, Quanguo Zhang

**Affiliations:** 1 Faculty of Geographical Science, State Key Laboratory of Earth Surface Processes and Resource Ecology, Zhuhai Joint Innovative Center for Climate-Environment-Ecosystem and Key Laboratory of Urban Climate and Ecodynamics, Future Earth Research Institute, Beijing Normal University, Beijing, 100875/Zhuhai, 519087 China; 2 School of Atmospheric Sciences, Sun Yat-Sen University, Guangzhou, 519082 Guangdong China; 3 Commonwealth Scientific and Industrial Research Organization, Oceans and Atmosphere, Private Bag 1, Aspendale, Victoria, 3195 Australia

## Abstract

Carbon allocation is one of the most important physiological processes to optimize the plant growth, which exerts a strong influence on ecosystem structure and function, with potentially large implications for the global carbon budget. However, it remains unclear how the carbon allocation pattern has changed at global scale and impacted terrestrial carbon uptake. Based on the Community Atmosphere Biosphere Land Exchange (CABLE) model, this study shows the increasing partitioning ratios to leaf and wood and reducing ratio to root globally from 1979 to 2014. The results imply the plant optimizes carbon allocation and reaches its maximum growth by allocating more newly acquired photosynthate to leaves and wood tissues. Thus, terrestrial vegetation has absorbed 16% more carbon averagely between 1979 and 2014 through adjusting their carbon allocation process. Compared with the fixed carbon allocation simulation, the trend of terrestrial carbon sink from 1979 to 2014 increased by 34% in the adaptive carbon allocation simulation. Our study highlights carbon allocation, associated with climate change, needs to be mapped and incorporated into terrestrial carbon cycle estimates.

## Introduction

Significant advances have been made in quantifying the impacts of climate change on terrestrial carbon cycling at local, regional, and global scales, thereby highlighting the role of climate in determining the terrestrial carbon cycle^1–4^. Numerous studies have demonstrated significant physiological adaptations and the regulation of terrestrial vegetation in response to climate change^5, 6^, where plants can mitigate negative impacts via physiological regulation to promote their growth. The impacts of climate change induced plant physiological changes on the carbon sink are greater than the direct effect of climate change on the carbon sink^7^. However, few studies have quantified the impacts of adaptive physiological response to a changing climate on global carbon cycle.

Carbon allocation is a critical physiological process where the products of photosynthesis are shifted between respiration and biomass production, ephemeral and long-lived tissues, and aboveground and belowground components. Changes in carbon allocation affect the growth of individual plants^8^ and terrestrial biogeochemistry via influences on litter quality and decomposition rates, carbon and nitrogen sequestration, and plant–atmosphere gas exchange^9, 10^. Theoretically, plants preferentially allocate carbon to acquire the resource that most limits their growth^11^, which is one of the most important mitigation processes for reducing the impacts of unfavorable environments. Evidence from field studies indicates that plants allocate relatively more carbon to shoots under light limitation and to roots under water and/or nutrient limitation^12–15^. For example, Lapenis *et al*.^16^ found that spruce forests allocate more carbon to roots to compensate for the root consumption caused by soil acidification and climate warming due to the earlier onset of the growing season.

Significant advances have been made in understanding carbon allocation at local scales^17, 18^, but few studies have focused on the contribution of carbon allocation to the global terrestrial carbon sink. In this study, we first used the Community Atmosphere Biosphere Land Exchange (CABLE) model investigate how the carbon allocation varies with climate change; then two modeling experiments were conducted to quantify the contribution of changes in carbon allocation to the terrestrial carbon cycle in response to climate changes.

## Data and Methods

The CABLE model version 2 is a global land surface model, which has been integrated with the CASACNP model to simulate the energy, water, carbon, nitrogen, and phosphorus cycles in terrestrial ecosystems^19–22^. The CABLE model can simulate the carbon allocation with two schemes using a daily time step: (1) a fixed coefficient scheme (FIX) and (2) a resource limitation scheme (RES). Both the FIX and RES schemes separate the growing season into three phases: maximal leaf growth, steady growth phase, and leaf senescence phase (Table 1), where the three phenophases were determined based on estimates from satellite-based observations^23^. In the FIX scheme, a set of constant fractions are used to determine the carbon allocation from net primary productivity to leaves, wood tissues, and roots, where the fractions differ in various phenological phases in deciduous biomes (Table 1)^19^. In the RES scheme, the allocation coefficients are adjusted dynamically according to the resource (i.e., light, water and nitrogen) that is most limiting on growth based on Friedlingstein *et al*.^9^. Note that evergreen biomes are assumed to be maintained at phenological phase 2 throughout the year (Table 1).

**Table 1:** Carbon allocation parameters in two carbon allocation schemes in CABLE.

Vegetation types	Fixed coefficient (FIX) model			Resource limitation (RES) model		
	Leaf (aleaf_FIX_)	Wood (awood_FIX_)	Root (aroot_FIX_)	Leaf (aleaf_RES_)	Wood (awood_RES_)	Root (aroot_RES_)
**1: Maximal leaf growth**						
Woody biomes	0.80	0.10	0.10	0.80	0.10	0.10
Non-woody biomes	0.80	—	0.20	0.80	—	0.20
**2: Steady growth phase**						
ENF	0.25	0.40	0.35	Dynamic allocation coefficients regulated by the available light, water, and nitrogen (see Data and Methods).		
EBF	0.20	0.35	0.45			
DNF	0.40	0.30	0.30			
DBF	0.35	0.25	0.40			
SHB	0.35	0.25	0.40			
C3	0.35	—	0.65			
C4	0.35	—	0.65			
TDR	0.50	0.10	0.40			
CROP	0.50	—	0.50			
**3: Leaf senescence phase**						
Woody biomes	0	awood_FIX_p2_/(awood_FIX_p2_ + aroot_FIX_p2_)	aroot_FIX_p2_/(awood_FIX_p2_ + aroot_FIX_p2_)	0	awood_RES_p2_/(awood_RES_p2_ + aroot_RES_p2_)	aroot_RES_p2_/(awood_RES_p2_ + aroot_RES_p2_)
Non-woody biomes	0	—	1.0	0	—	1.0

ENF: evergreen needleleaf forest; EBF: evergreen broadleaf forest; DNF: deciduous needleleaf forest; DBF: deciduous broadleaf forest; SHB: shrub; C3: C3 grass; C4: C4 grass; TDR: tundra; CROP: C3 crop. ENF, EBF, DNF, DBF, SHB, and TDR are woody biomes. C3, C4, and CROP are non-woody biomes. awood_FIX_p2_ and aroot_FIX_p2_ denote the fixed carbon allocation ratios to wood tissues and roots in the steady growth phase (phase 2). awood_RES_p2_ and aroot_RES_p2_ denote the resource limitation carbon allocation ratios to wood tissues and roots in the steady growth phase (phase 2).

The RES scheme uses the method proposed by Friedlingstein *et al*.^9^. For woody biomes, the allocation coefficients for leaves (*aleaf*), woody tissues (*awood*), and roots (*aroot*) are expressed as follows.

1 $$aroot=3{r}_{0}\frac{L}{L+2\,{\rm{\min }}(W,N)}$$

2 $$awood=3{s}_{0}\frac{{\rm{\min }}(W.N)}{2L+\,{\rm{\min }}(W,N)}$$

3 $$aleaf=1-awood-aroot$$

The allocation coefficients for non-woody biomes are given by:

4 $$aroot=3{r}_{0}\frac{L}{L+2\,{\rm{\min }}(W,N)}$$

5 $$aleaf=1-aroot$$

where min(*W, N*) is the minimum availability of water (*W*) and nitrogen (*N*), and *r*_*0*_ = *s*_*0*_ = 0.3. Carbon allocation for leaf, wood and root and light, water, nitrogen availabilities have no units and vary from 0 to 1.

Light availability (*L*) is calculated by:

6 $$L=\,{\rm{\min }}[\max ({e}^{-0.5LAI}),1]$$

where *LAI* is the leaf area index.

Water availability (*W*) is calculated by:

7 $$W=\,\min [\max (\sum _{i=1}^{n}f{r}_{i}\frac{{S}_{Wi}-{S}_{wilt}}{{S}_{field}-{S}_{wilt}}),1]$$

where *fr*_*i*_ is the fraction of roots present in the *i*th soil layer, *S*_*wi*_ is the volumetric soil moisture content in the *i*th soil layer, and *S*_*field*_ and *S*_*wilt*_ represent the field capacity soil moisture contents and wilting point, respectively.

Nitrogen availability (*N*) is calculated by combining the temperature (*T*_*s*_) and moisture (*W*_*s*_) limitations^9^, where the temperature limitation is calculated based on a standard *Q*_*10*_ equation^24^ and the moisture limitation is calculated as described by equation (7):

8 $$N={T}_{s}\,\times {W}_{s}$$

9 $${T}_{s}={{Q}_{10}}^{(\frac{{\sum }_{i=1}^{n}f{r}_{i}{T}_{soili}-30}{10})}$$

where *T*_*soili*_ is the temperature of the *i*th (*i* = 6) soil layer (°C) and *Q*_*10*_ = 2.

The carbon allocation coefficients will change the simulated carbon fluxes and pools through the following governing equations. The carbon allocation to plan components will change the carbon pools (equation 10). The leaf biomass controls the leaf area index and then changes the leaf photosynthesis (equation 11).

10 $$\frac{d{C}_{j}}{dt}=NPP\times {a}_{j}-{C}_{j}\times {\tau }_{j},j=leaf,wood\,or\,root$$

11 $$LAI={C}_{leaf}\times SLA$$

where *C* denotes pool size in g C m^−2^ and *τ* turnover rate in day^−1^, both with one subscript, *j* for plant pool. *a*_*j*_ is the fraction of net primary productivity (*NPP*) allocated to plant pool *j*. *SLA* is the specific leaf area in m^2^ g C^−1^.

We used the hourly Modern Era Retrospective-Analysis for Research and Applications (MERRA)^25^ dataset to drive the CABLE model, with the following parameters: the near surface air temperature (Tair), rainfall rate (Rain), incident shortwave radiation (Swd), incident longwave radiation (Lwd), specific humidity (Qair), wind speed (Wind) and pressure (Ps) during 1979–2014. MERRA is a NASA reanalysis for the satellite era, which uses a new version of the Goddard Earth Observing System Data Assimilation System Version 5 at a resolution of 0.5° latitude by 0.67° longitude. The annual mean global surface CO_2_ concentration during 1850–2014 was obtained from a combination of ice core records and atmospheric observations^26^. The fixed spatial distribution of the dominant plant functional types for the year 2005 was based on (Fig. S1)^27^.

We performed two modeling experiments to quantify the contribution of carbon allocation to the terrestrial carbon sink: (1) CABLE with the FIX carbon allocation scheme (FIX simulation), and (2) CABLE with the RES carbon allocation scheme (RES simulation). In both simulations, CABLE was run with carbon, nitrogen, and phosphorus cycles. The canopy LAI was predicted according to the time-varying leaf biomass. Ecosystem disturbance and land cover changes were not considered in this study. To obtain the initial sizes of all the carbon, nitrogen, and phosphorus pools, a spin-up of 720 years was conducted by recycling 20 times of the the meteorological variables derived from the MERRA dataset from 1979 to 2014 with an atmospheric CO_2_ concentration of 280.0 ppm (i.e., 1850 level). The model was then restarted from the spin-up conditions in 1850 and run for the period 1850–1979 (i.e., 130 years) by repeatedly using the MERRA climate from 1979 to 2014 and historical CO_2_ observations. The two modeling experiments were restarted from the conditions in 1979 and simulated for the period 1979–2014 under forcing with the MERRA climate from 1979 to 2014 and atmospheric CO_2_ concentration observations. The differences in the carbon allocation ratios and terrestrial carbon sink between the RES and FIX simulations represented the effects of carbon allocation changes.

## Results

**Figure 1:**
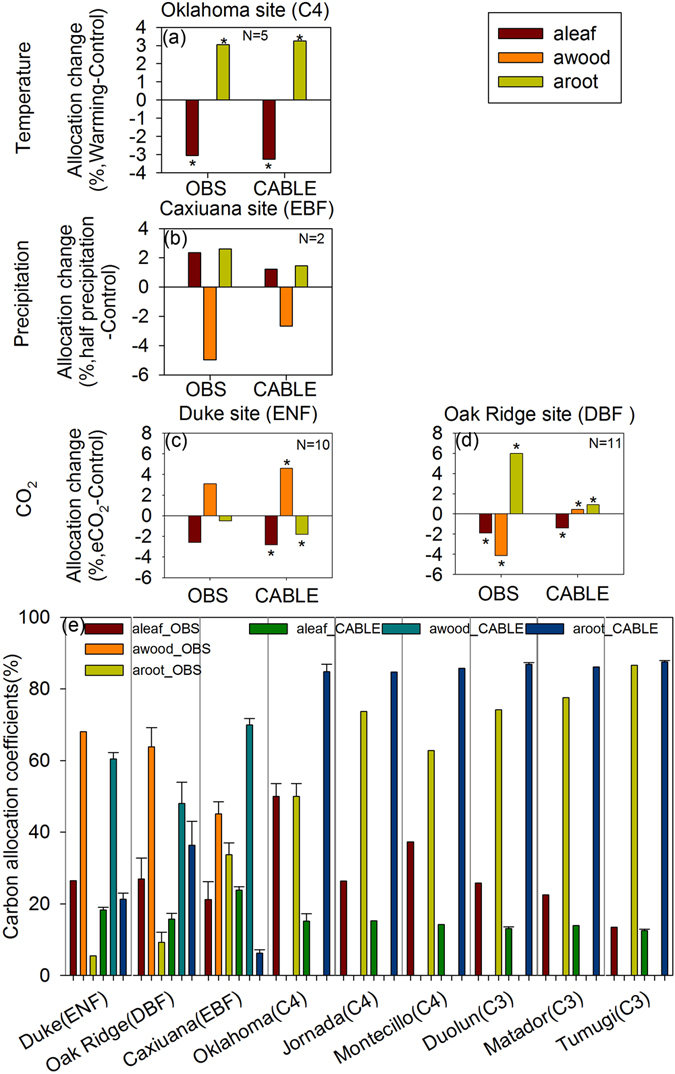
Comparison of carbon allocation coefficients to leaves (aleaf), woody tissues (awood), and roots (aroot) between observations (OBS) and CABLE model with the resource limiation allocation scheme at warming (**a**), precipitation reduction (**b**) and elevated atmospheric CO_2_ (eCO_2_, **c**,**d**) sites and and natural sites (**e**). ENF: evergreen needleleaf forest; EBF: evergreen broadleaf forest; DBF: deciduous broadleaf forest; C3: C3 grass; C4: C4 grass. ^*^Significant change (*p* < 0.05).

The CABLE with the RES carbon allocation scheme showed the potential in reproducing the realistic changes of carbon allocation coefficients to leaves (aleaf_RES_), wood (awood_RES_) and root (aroot_RES_) with environmental changes (e.g., warming, elevated atmospheric CO_2_, and drought) (Fig. 1 and Table S1). Both observation and simulation of CABLE model showed that the warming experiment at the C4 grass site in Oklahoma significantly increased aroot_RES_ (Fig. 1a). The drought experiment at evergreen broadleaf forest in Caxiuana site showed that forest will increase aroot_RES_ and aleaf_RES_ and decrease awood_RES_ under drought condition (Fig. 1b). The CABLE model correctly simulated the effects of the elevated atmospheric CO_2_ (eCO_2_) on carbon allocation at Duke site (Fig. 1c). But, the eCO_2_ induced decreasing of awood_RES_ at Oak Ridge site was not captured by CABLE model (Fig. 1d).

The CABLE model with the RES carbon allocation scheme produced reasonable pattern of carbon allocation of forest and grass (Fig. 1e). The forest allocated the largest fractions of carbon to wood. The grass allocated more carbon to root than leaf, except for C4 grass at Oklahoma site. The discrepancies of carbon allocation between observations and the simulations of CABLE model were also found. For example, CABLE model underestimated the awood of deciduous broadleaf forest at Oak Ridge site and overestimated the awood of evergreen broadleaf forest at Caxiuana site. CABLE model overestimated the aroot of grass, especially at C4 grass sites.

**Figure 2:**
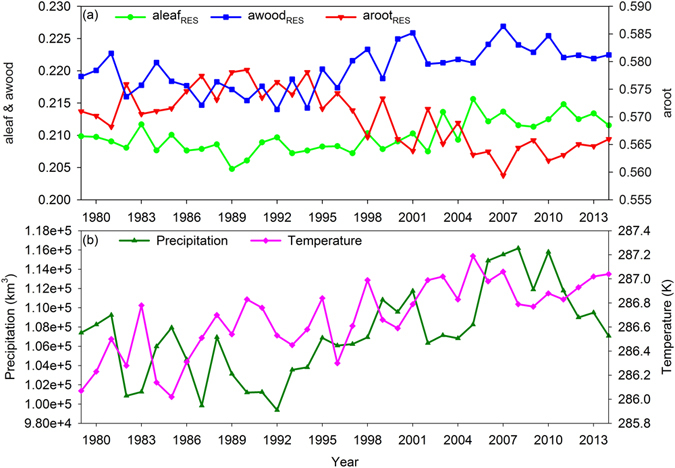
Estimated carbon allocation coefficients (**a**), and precipitation and temperature (**b**) from 1979 to 2014. The global mean annual carbon allocation to leaves (aleaf_REF_), wood (awood_REF_), and roots (aroot_REF_) were simulated by CABLE using the resource limitation (RES) model (**a**). The annual total precipitation and mean temperature of the plant-covered area were calculated from MERRA data (**b**).

Terrestrial vegetation optimized the carbon allocation among three components, i.e. roots, wood tissues, and leaves, with changes in the environmental variables. The average aleaf_RES_ and awood_RES_ in the RES simulation increased significantly from 1979 to 2014 globally (Fig. 2), and a lower fraction of carbon was partitioned to the roots with increasing precipitation and temperature (Fig. 2 and Fig. S2). Compared with the FIX carbon allocation scheme, the RES scheme had a higher carbon partition ratio and trends in leaves and wood (Tables S2 and S3). For example, the global mean allocation ratios to leaves and wood according to the RES simulation were 0.21 ± 0.003 and 0.22 ± 0.003, which were larger than those in the FIX simulation (Table S2).

**Figure 3:**
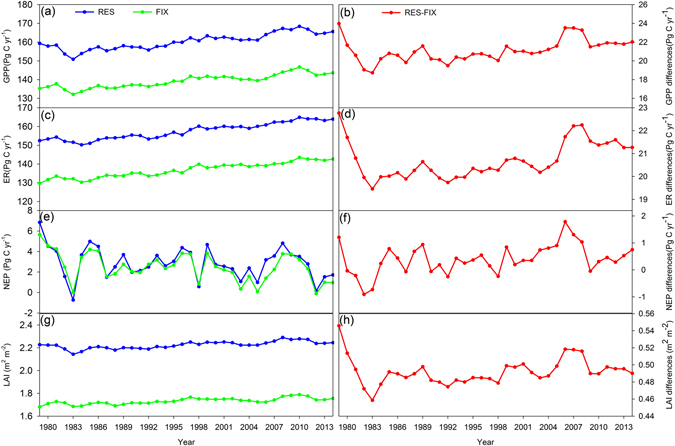
Estimated gross primary production (GPP), ecosystem respiration (ER), net ecosystem productivity (NEP), and leaf area index (LAI) from 1979 to 2014. RES and FIX refer to the four variables from CABLE with resource limitation and fixed coefficient carbon allocation models, respectively. RES–FIX refers to their differences.

**Figure 4:**
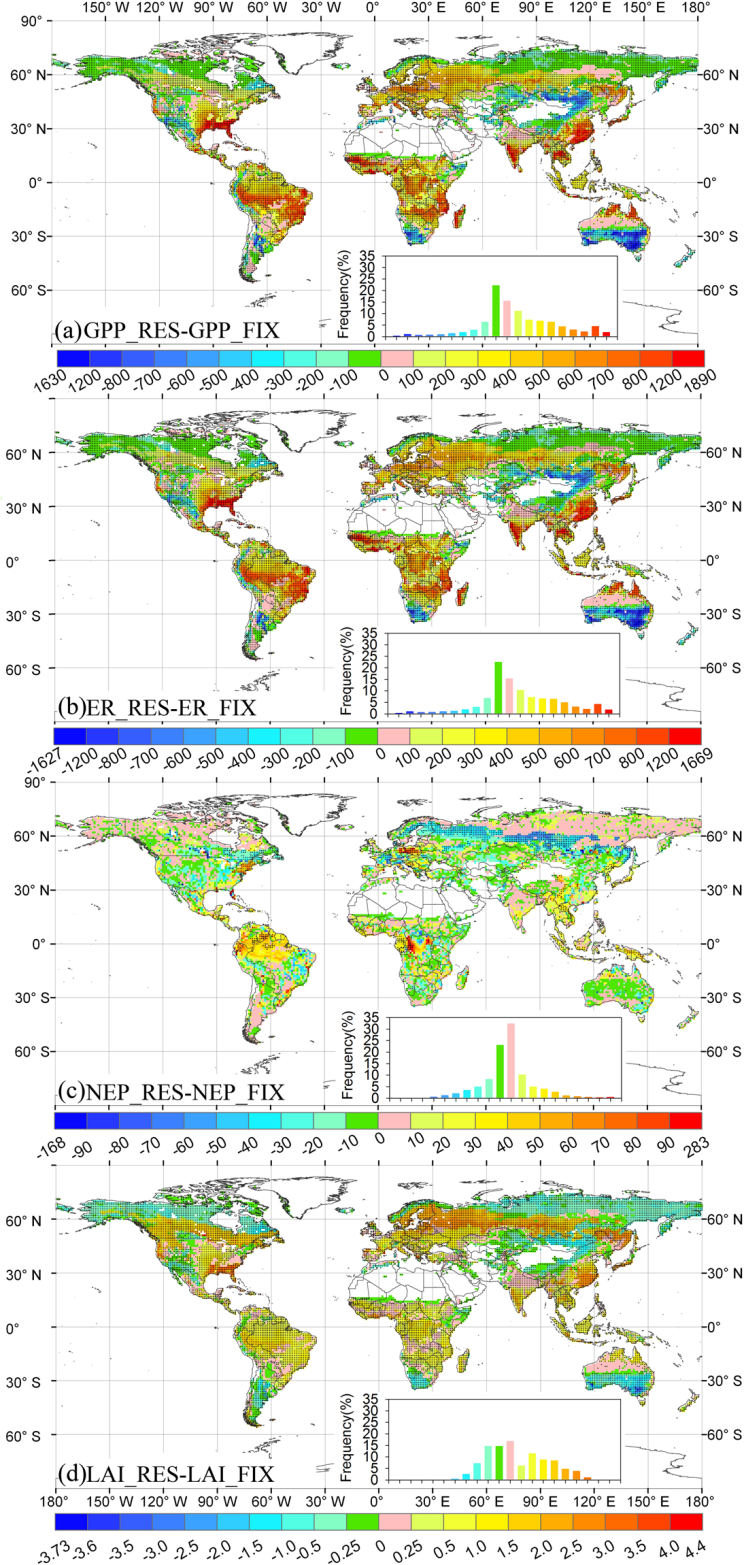
Spatial distribution of the differences in gross primary production (GPP, g C m^−2^ yr^−1^), ecosystem respiration (ER, g C m^−2^ yr^−1^), net ecosystem productivity (NEP, g C m^−2^ yr^−1^), and leaf area index (LAI) between CABLE using the resource limitation (RES) and fixed coefficient (FIX) carbon allocation models. The inset shows the frequency distribution of the corresponding differences. Stippling shows regions where the differences are statistically significant at the 95% level. The maps were created by the ArcMap 9.3.

The adaptive carbon allocation (i.e., RES carbon allocation) increased the magnitude of the terrestrial carbon sink. The gross primary production (GPP) and ecosystem respiration (ER) were higher in the RES simulation than the FIX simulation. However, the increase in ER (20.70 Pg C yr^−1^) was less than that in GPP (21.102 Pg C yr^−1^), and thus the adaptive carbon allocation enhanced the terrestrial ecosystem sink (Fig. 3a–f). On average, the RES simulation estimated a global net ecosystem productivity (NEP) of 2.88 Pg C yr^−1^, which was 16% larger than that in the FIX simulation (Table S2). Globally, 62% of the areas had larger GPP estimates in the RES simulation, and lower GPP values were only found over tundra as well as parts of the shrublands and C3 grasslands (Fig. 4a). The difference in ER between the RES and FIX simulations had a similar spatial pattern to that obtained for the GPP (Fig. 4a,b). The difference in NEP exhibited a positive change in the majority of the tundra and evergreen broadleaf forest region (Fig. 4c). The largest positive change in the difference in NEP was found in evergreen broadleaf forest, with a value of 0.442 Pg C yr^−1^, and the lowest negative change was in evergreen needleleaf forest, with a value of –0.157 Pg C yr^−1^ (Fig. 4c and Table S2).

**Figure 5:**
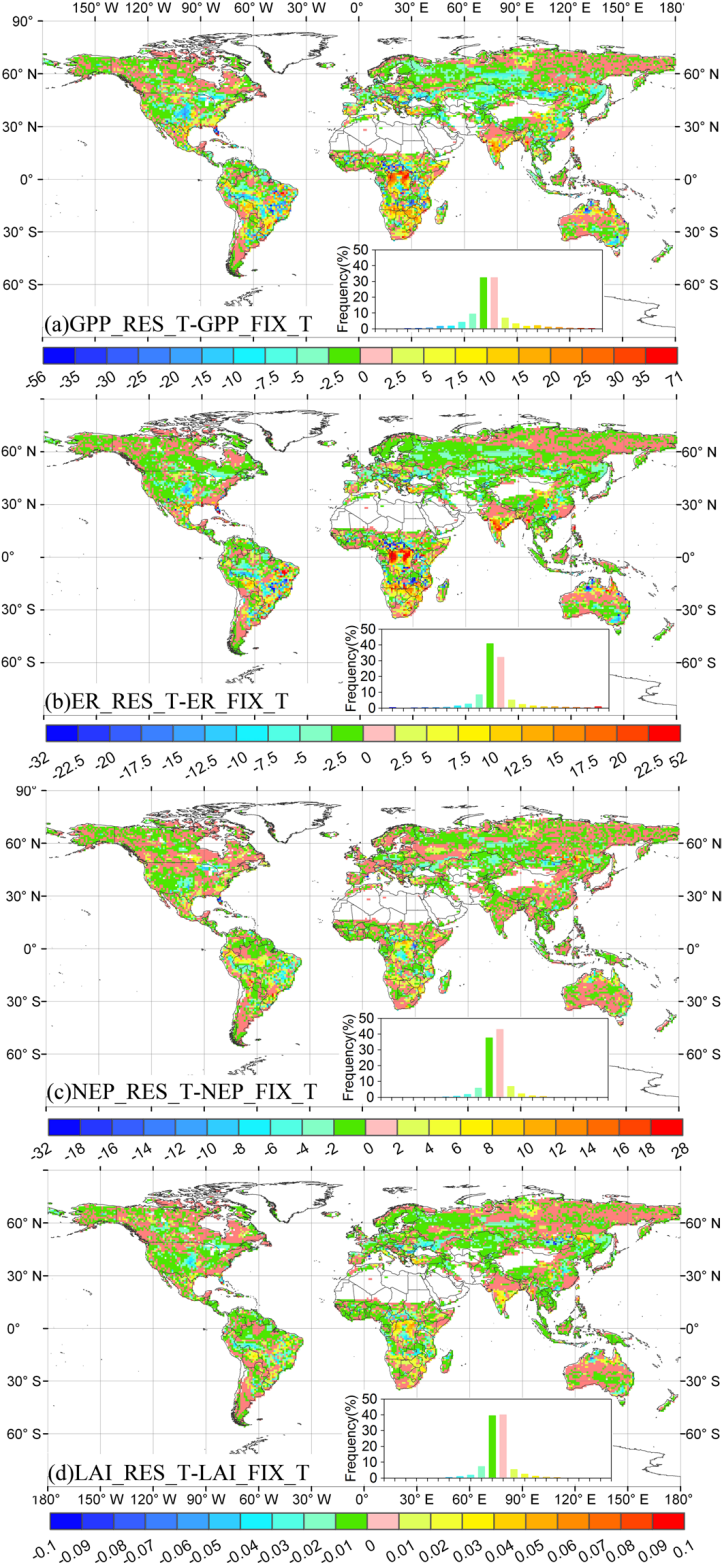
Differences in the trends in gross primary production (GPP, g C m^−2^ yr^−2^), ecosystem respiration (ER, g C m^−2^ yr^−2^), net ecosystem productivity (NEP, g C m^−2^ yr^−2^), and leaf area index (LAI) between CABLE using the resource limitation (RES_T) and fixed coefficient (FIX_T) carbon allocation models during 1979–2014. The insets show the frequency distributions of the corresponding trends. The maps were created by the ArcMap 9.3.

Adaptive carbon allocation increased the trend in the terrestrial carbon sink from 1979 to 2014. Compared with the FIX simulation, the trends in GPP, ER, and NEP increased by 0.052 Pg C yr^−2^ (17%), 0.032 Pg C yr^−2^ (9%), and 0.020 Pg C yr^−2^ (34%), respectively, in the RES simulation (Table S3 and Fig. 3a–f). This means that with adaptive carbon allocation parameterization, RES simulation would be expected to increase the fluxes faster than FIX simulation under climate change. The difference in the NEP trend obtained by the RES and FIX simulations was positive over 54% of the study area, which was larger than that in the GPP (50%) and ER (45%) (Fig. 5). The evergreen needleleaf forest contributed to the largest increase in the trend in the NEP, followed by evergreen broadleaf forest (Table S3).

Compared with the FIX simulation, the RES simulation obtained a larger LAI and allocation ratio to leaves (Figs 3g,h and 4d, and S3a), which contributed to the higher GPP, ER, and NEP. The differences in GPP and ER between the RES and FIX simulations were strongly correlated with the difference in LAI (R^2^ = 0.63 and 0.64, Fig. S4). The changes in NEP depended on the different responses of GPP and ER to the changes in LAI (Fig. 4). The differences in the trends in GPP, ER, and NEP obtained by the RES and FIX simulations had strong correlations with the differences in the trend in LAI (Fig. 5, S5, and S6). The differences in the trend in LAI obtained by the RES and FIX simulations were consistent with the differences in the trend in the allocation ratio to leaves in 73% of the study area (Fig. 5d and S5a).

## Discussion and Conclusions

**Figure 6:**
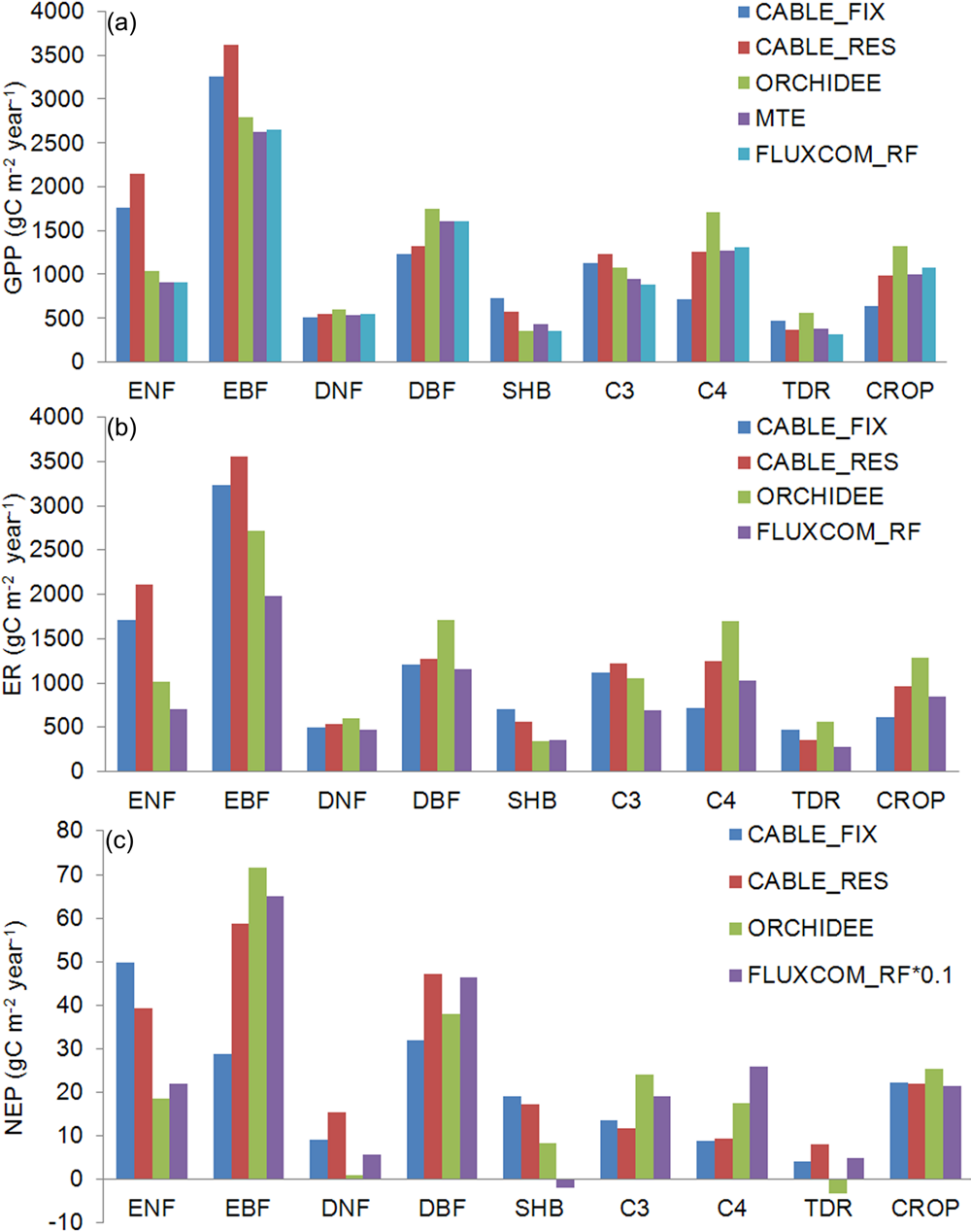
The estimated mean (**a**) gross primary production (GPP), (**b**) ecosystem respiration (ER) and (**c**) net ecosystem productivity (NEP) for different plant functional types by CABLE for the FIX (CABLE_FIX) and RES (CABLE_RES) simulations, ORCHIDEE model in the Trendy project (Sitch *et al*.^28^; Le Quéré *et al*.^29^), and random forests method in the FLUXCOM global carbon flux data set (FLUXCOM_RF) from Jung *et al*.^32^. The MTE GPP is from Beer *et al*.^30^ and Jung *et al*.^31^. ENF: evergreen needleleaf forest; EBF: evergreen broadleaf forest; DNF: deciduous needleleaf forest; DBF: deciduous broadleaf forest; SHB: shrub; C3: C3 grass; C4: C4 grass; TDR: tundra; CROP: C3 crop.

The GPP, ER and NEP simulations of CABLE model are comparable with those estimates derived by data-driven models and ORCHIDEE model^28–32^. The mean GPP for different plant functional types as calculated by CABLE with RES allocation scheme (Fig. 6a) is quite similar to those estimates of ORCHIDEE model^28, 29^, MTE method^30, 31^ and random forests method in the FLUXCOM global carbon flux data set (FLUXCOM_RF)^32^ except evergreen needle leaf forest and evergreen broadleaf forest. The mean ER estimates of CABLE and ORCHIDEE models are generally larger than the ER of FLUXCOM_RF method (Fig. 6b). The mean NEP estimates of CABLE model are close to those of ORCHIDEE model except evergreen needle leaf forest, deciduous needleleaf forest and shrub (Fig. 6c). The NEP of FLUXCOM_RF method is about ten times the NEP estimates of CABLE and ORCHIDEE models (Fig. 6c).

Despite significant advances in the past decade, major uncertainties remain concerning the processes and mechanisms related to the terrestrial ecosystem carbon sink^33^. Many previous studies have investigated the impacts of climate change^34^, but the physiological changes that occur in plants in response to climatic variability are also critical for understanding the carbon cycle^35, 36^. Our results show that the adaptive carbon allocation increased terrestrial ecosystem carbon sink by an average of 16% from 1979 to 2014, and results in a long-term increasing trend in the terrestrial carbon sink of 34% (Tables S2 and S3). From 1979 to 2014, the global precipitation and temperature exhibited an increasing trend, and plants could invest more carbon in leaves and wood tissues to enhance vegetation production as well as the terrestrial carbon sink at a global scale (Figs 2, 3, and S2)^37^. Precipitation, temperature, and light together controlled the trends in the carbon allocation ratios (Fig. S2). The spatial changes in carbon allocation ratios were controlled by the water and light conditions. For example, the high water and low light conditions in tropical forest determined the low root allocation and high wood and leaf allocation (Fig. S3).

The increased carbon allocation to wood and leaves enhanced the terrestrial carbon sink partly because of the different turnover rates of plant components. The turnover rate is fastest for leaves (0.15–0.77 yr^−1^)^38^, intermediate for roots (0.1 yr^−1^, 0.055–0.33 yr^−1^)^39, 40^, and they are both significantly faster than that for wood (0.01–0.05 yr^−1^, 0.012 yr^−1^)^41, 42^. Our results showed that the largest increases in the wood allocation ratio were obtained using the RES scheme compared with the FIX scheme, as well as significant decreases in the root allocation (Table S2). The turnover rate for plant components is directly correlated with the annual litter input, as well as the substrate for heterotrophic respiration^43^. The carbon allocated to wood tissues will be stored in terrestrial ecosystem for a long time, and reduce the magnitude of leaf and root litterfall.

The adaptive carbon allocation, which is associated with climate change, needs to be incorporated into future evaluations of the terrestrial carbon sink. Climate model predictions suggest severe and widespread droughts in the next 30–90 years over many land areas^44^. Moreover, the soil nitrogen availability will be changed significantly by increasing nitrogen deposition in the future^45^. These changes in environmental resources will directly impact the terrestrial carbon cycle, and indirectly alter the partitioning of assimilated carbon among the plant components^17, 46, 47^. Our study highlights the importance of evaluating the impacts of carbon allocation to the terrestrial carbon budget and there is an urgent need to incorporate these impacts in future studies. Furthermore, it is important to differentiate the impacts of various environmental changes on carbon allocation and investigate the dominant factors to understand the changes in carbon allocation.

The CABLE model has been validated and applied globally, but there are several uncertainties in the model, which need further improvement to investigate the impacts of carbon allocation on the carbon cycle. First, the empirical nitrogen availability equations are used to indicate soil nitrogen availability. Temperature and soil moisture are two most important variables controlling organic nitrogen mineralization and dominating soil nitrogen content. This study used these two empirical equations to simulate nitrogen mineralization and nitrogen availability. However, the method does not include nitrogen nitrification and denitrification processes, and decreases the ability to determine soil nitrogen content. Moreover, the empirical nitrogen model cannot indicate atmospheric nitrogen deposition, which strongly regulates the soil nitrogen availability in eastern USA, Europe, China and India^45^. Thus, the empirical nitrogen availability may result in large uncertainties in terms of the model’s performance. Moreover, the CABLE model cannot indicate the limitation of phosphorus relative to the carbon allocation. Phosphorus limitation is a primary constraint on plant growth in tropical regions^48, 49^. Baribault *et al*.^50^ showed that the wood growth rate in tropical forests is positively correlated with soil phosphorus availability. Therefore, the CABLE model will probably overestimate the wood allocation in tropical regions without integrating phosphorus limitation into the carbon allocation model. Therefore, future studies should incorporate process-based nitrogen and phosphorus limitations into the allocation model.

In addition, theoretically, adaptive carbon allocation can retard the environmental stresses and accelerate plant growth. However, in this study, the adaptive carbon allocation did not always show larger GPP or NEP compared those of model using fix carbon allocation ratio. Especially, over the drought and cold areas with limited soil moisture and nitrogen, GPP simulations derived by dynamic carbon allocation model are lower than those of fix carbon allocation model (Fig. 4a). The main causes lie in incomplete root function in the current models, not only in CABLE. Current ecosystem models make fine root grow by allocating carbon to fine roots. However, the absorbed water and nutrient by plants are independent on the fine root biomass, only determined by soil water and nutrient availability^51^. This imperfect model structure conceals the positive impacts of carbon allocation on vegetation growth. Therefore, processes-based soil water and nutrient absorbed model, associated with dynamic fine root growth, needs to be incorporated into the current ecosystem models in order to adequately assess the role of carbon allocation in the global carbon cycle.

In conclusion, we simulated the possible changes in the global carbon allocation patterns and quantified the effects of carbon allocation changes on the terrestrial carbon sequestration potential. The CABLE model with the RES carbon allocation scheme showed that more carbon can be absorbed by the terrestrial ecosystem. The dynamic carbon allocation significantly changed the carbon fluxes and plant structures (e.g., LAI). These changes are the indirect effects of climate change on the terrestrial ecosystem and they could lead to profound feedback in the climate system. Meanwhile, the resource limitation carbon allocation model is a kind of representative model that adopted by several terrestrial ecosystem models, such as CTEM^52^, ORCHIDEE^53^, and aDGVM^54^. The results imply the plant adjusts carbon allocation and reaches its maximum growth by allocating more newly acquired photosynthate to leaves and wood tissues. The dynamic carbon allocation by plants enhances the terrestrial carbon sink (Fig. 3f). So the findings in this study are generic for studies based on resource limitation carbon allocation model. Thus, the feedback between dynamic carbon allocation and climate change should be considered in estimates of the terrestrial carbon cycle.

## Electronic supplementary material

Supporting Information
