# Supplementary material for: Adaptive Carbon Allocation by Plants Enhances the Terrestrial Carbon Sink
Source: Sci Rep. 2017 Jun 13;7:3341. doi: 10.1038/s41598-017-03574-3 (PMC5469799; doi:10.1038/s41598-017-03574-3)
Supplement: Supplementary file 1 — Supporting Information [file 41598_2017_3574_MOESM1_ESM.doc]

*Scientific Reports*

Supporting Information for

**Adaptive Carbon Allocation by Plants Enhances the Terrestrial Carbon Sink**

**Jiangzhou Xia^1^, Wenping Yuan^1,2*^, Ying-Ping Wang^3^, Quanguo Zhang^1^**

^1^Faculty of Geographical Science, State Key Laboratory of Earth Surface Processes and Resource Ecology, Zhuhai Joint Innovative Center for Climate-Environment-Ecosystem and Key Laboratory of Urban Climate and Ecodynamics, Future Earth Research Institute, Beijing Normal University, Beijing 100875/Zhuhai 519087, China

^2^School of Atmospheric Sciences, Sun Yat-Sen University, Guangzhou 519082, Guangdong, China,

^3^Commonwealth Scientific and Industrial Research Organization, Oceans and Atmosphere, Private Bag 1, Aspendale, Victoria 3195, Australia

*Corresponding author: Wenping Yuan ([yuanwpcn@126.com)](mailto:email@address.edu))

**Contents of this file**

Figures S1 to S6

Tables S1 to S3


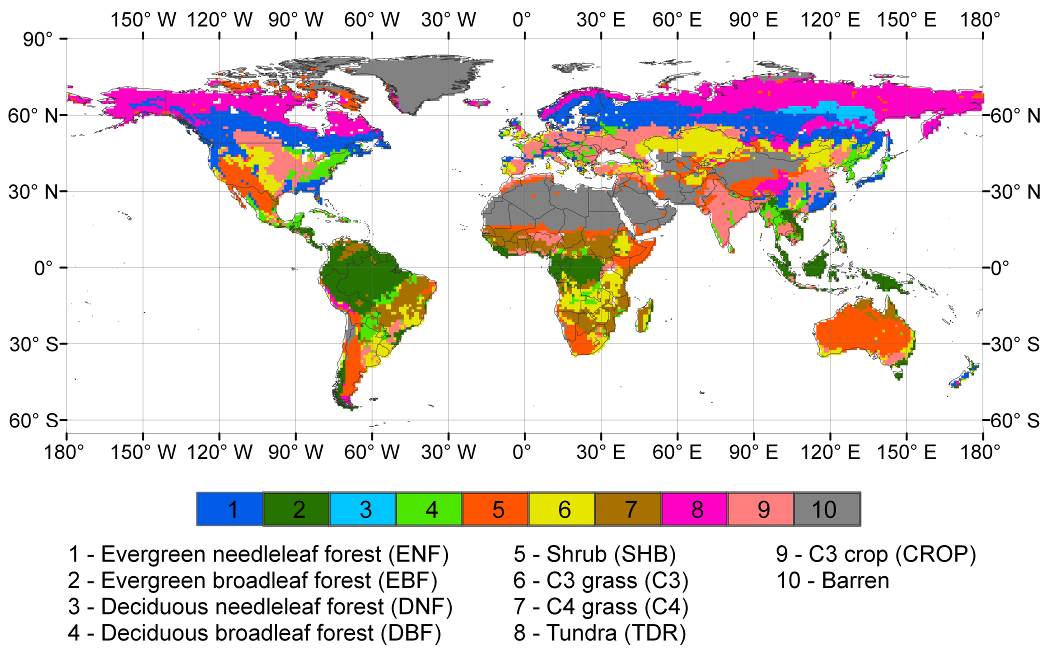


**Figure S1**. Global distribution of plant functional types. The maps were created by the ArcMap 9.3.


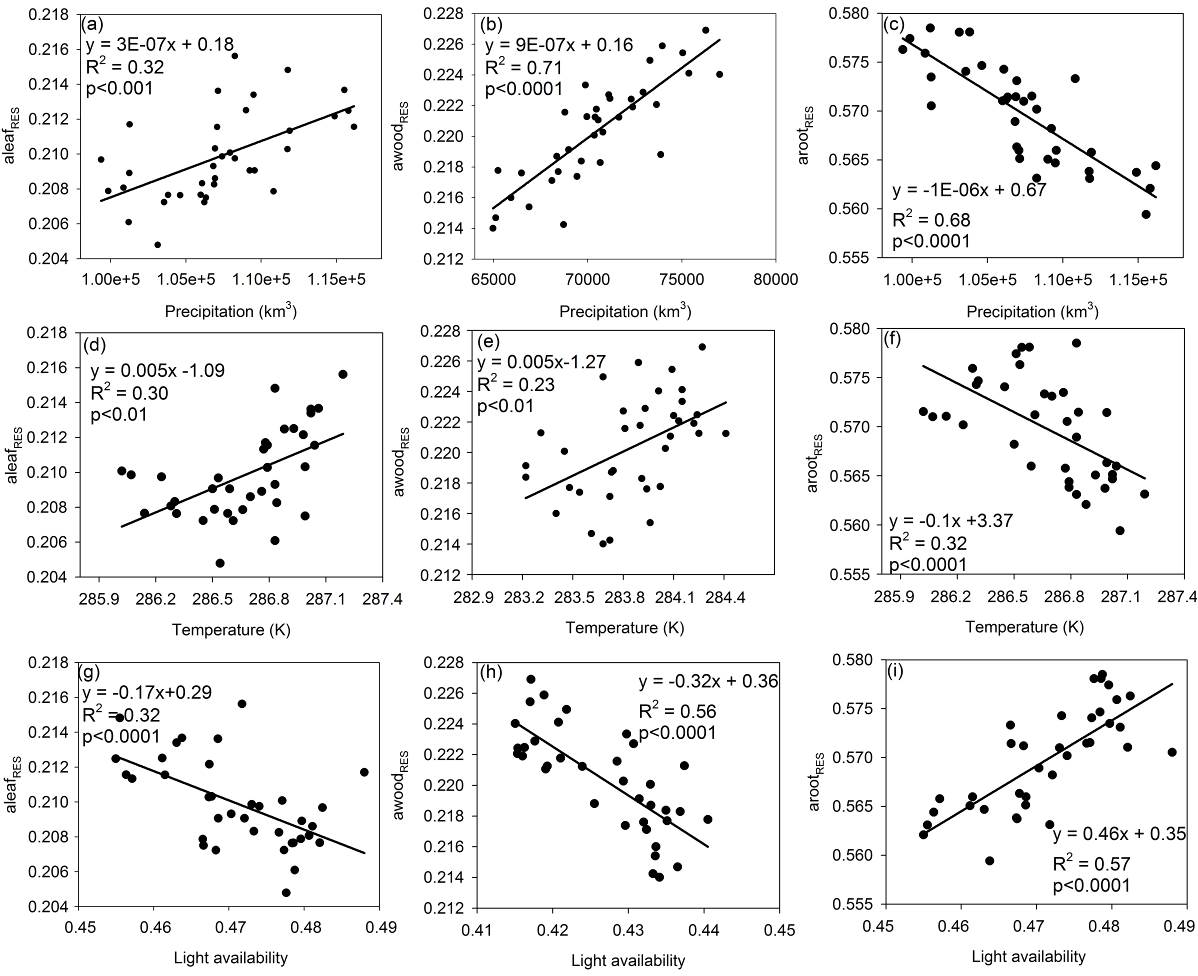


**Figure S2**. Relationships between global means of the resource limitation carbon allocation ratios and environmental variables (i.e., precipitation, temperature, and light availability) from 1979 to 2014. For the carbon allocation to wood (awood_RES_), the values were calculated only from the area of wood biomes (b, e, and h).


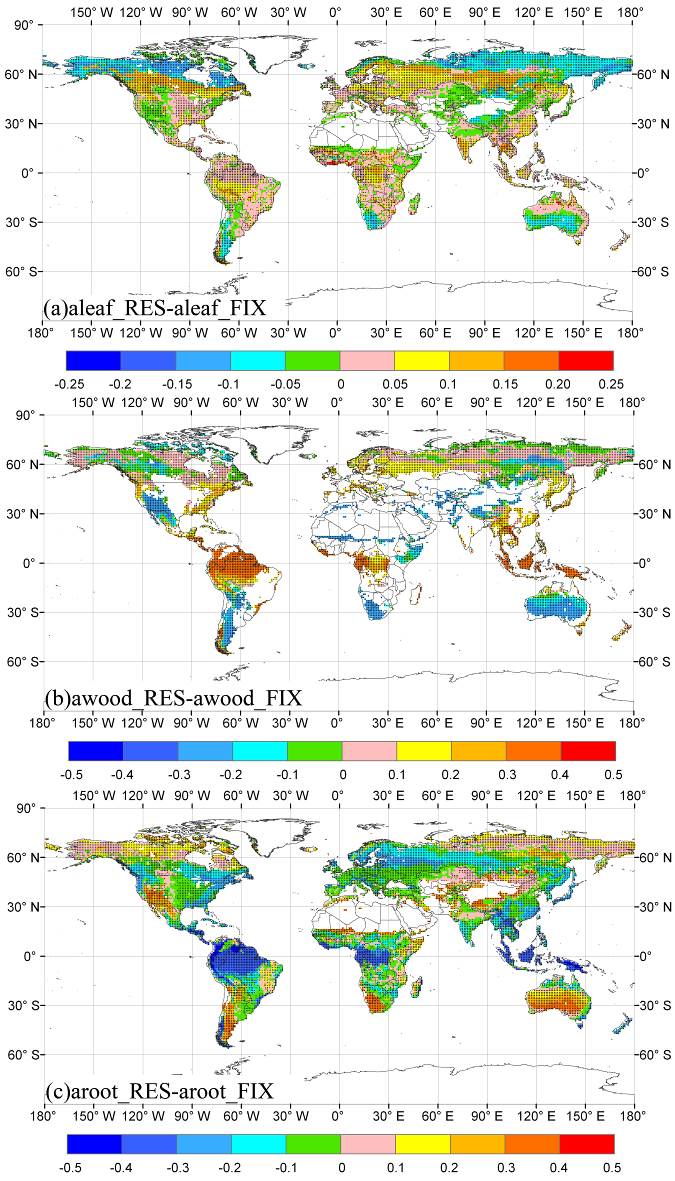


**Figure S3**. Spatial distribution of the differences in carbon allocation to leaves (aleaf), wood (awood), and roots (aroot) using CABLE with resource limitation (RES) and fixed coefficient (FIX) carbon allocation models. Stippling shows regions where the differences are statistically significant at the 95% level. The maps were created by the ArcMap 9.3.


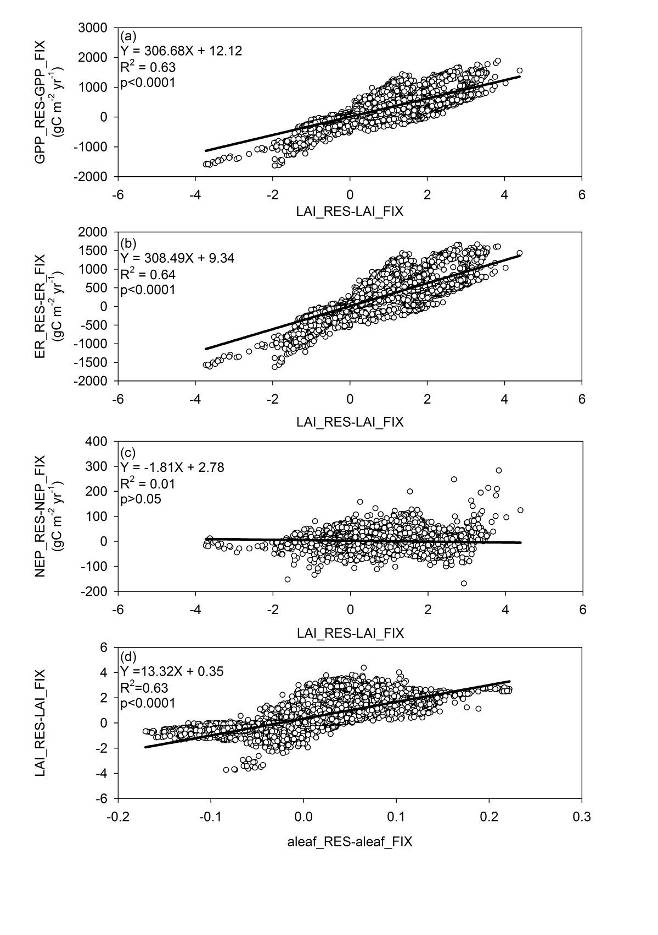


**Figure S4**. Correlations between the differences in gross primary production (GPP) (a), ecosystem respiration (ER) (b), and net ecosystem productivity (NEP) (c) with the leaf area index (LAI) in simulations using the resource limitation (RES) and fixed coefficient (FIX) carbon allocation models. (d) Correlations between the difference in LAI and the carbon allocation coefficient to leaves (aleaf).


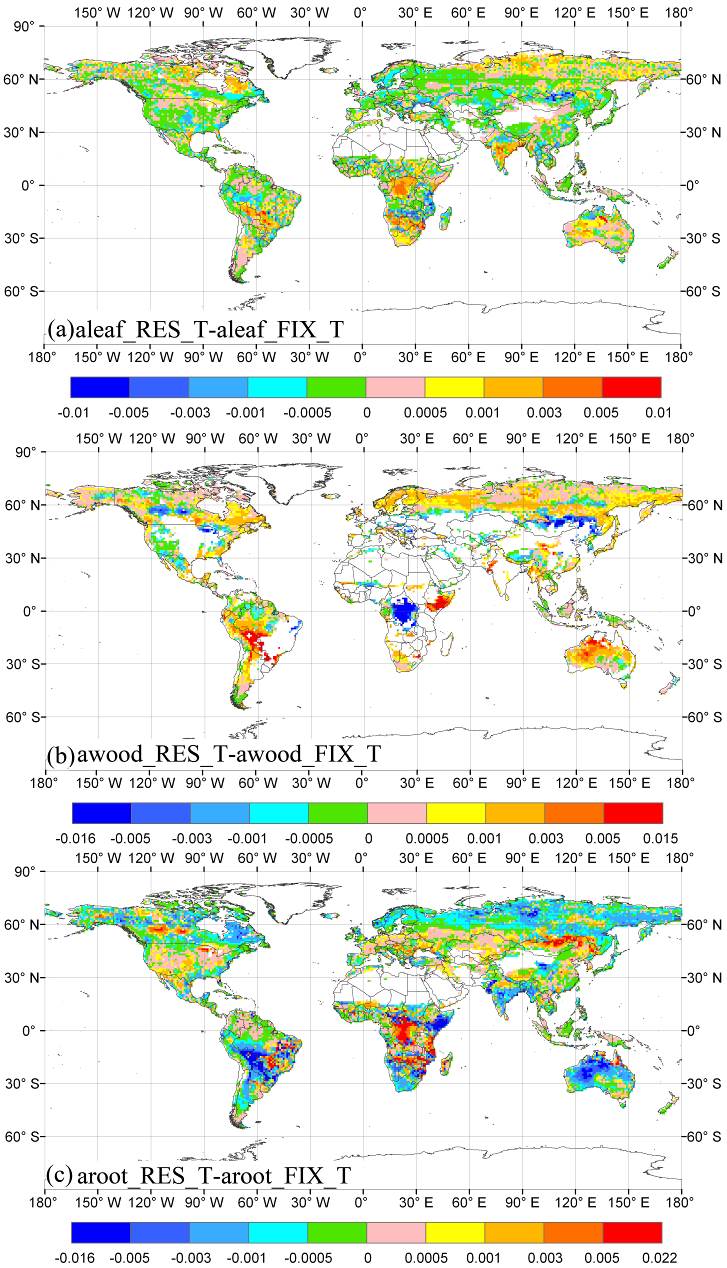


**Figure S5**. Differences in the trends in carbon allocation to leaves (aleaf), wood (awood), and roots (aroot) using CABLE with the resource limitation (RES_T) and fixed coefficient (FIX_T) carbon allocation models during 1979–2014. The maps were created by the ArcMap 9.3.


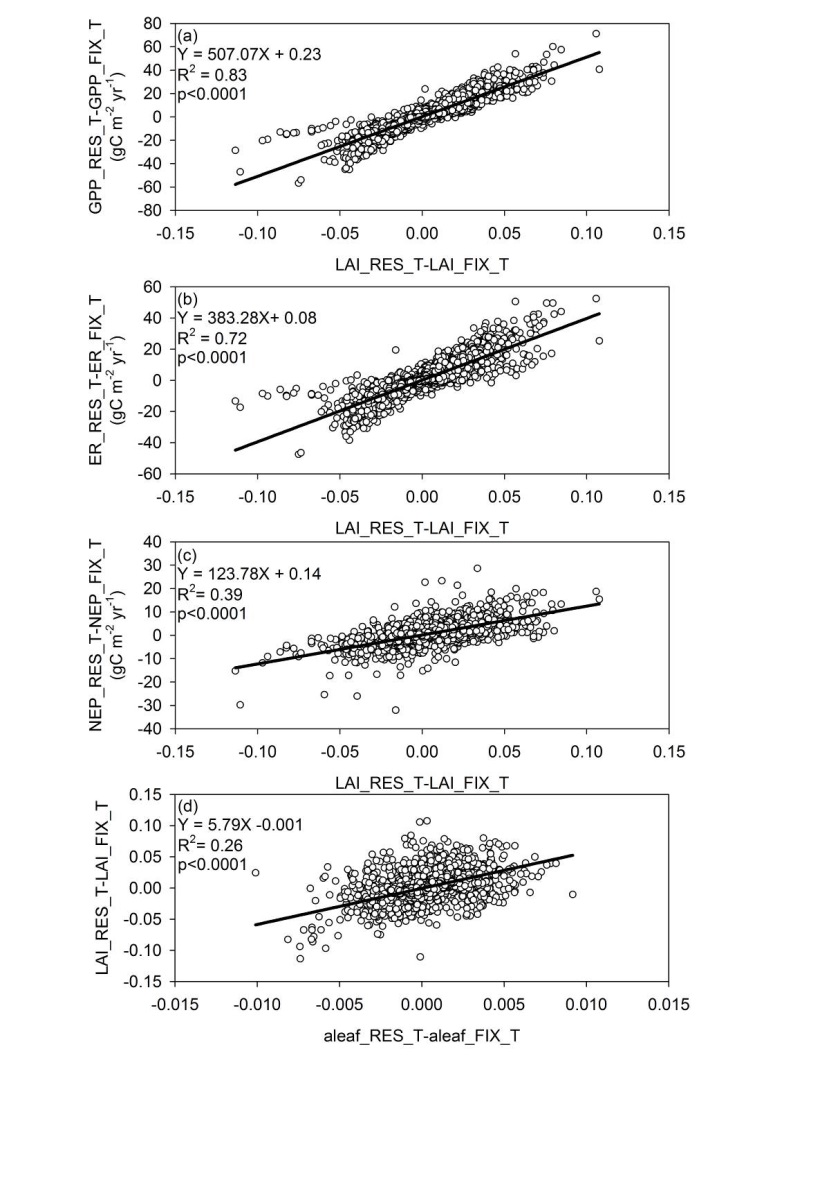


**Figure S6**. Correlations between the differences in the trends in gross primary production (GPP) (a), ecosystem respiration (ER) (b), and net ecosystem productivity (NEP) (c) with the leaf area index (LAI) simulated using the resource limitation (RES) and fixed coefficient (FIX) carbon allocation models. (d) Correlations between the differences in the trend in LAI and the carbon allocation coefficient to leaves (aleaf).

**Table S1**. The information of carbon allocation validation sites.

| Site name | Longitude | Latitude | Plant functional types | References |
| --- | --- | --- | --- | --- |
| Duke | -79.08 | 35.97 | Evergreen needleleaf forest | De Kauwe et al., 2014 |
| Oak Ridge | -84.33 | 35.9 | Deciduous broadleaf forest | De Kauwe et al., 2014 |
| Caxiuana | -51.45 | -1.7167 | Evergreen broadleaf forest | Doughty et al., 2015;  da Costa et al., 2013 |
| Oklahoma | -97.5167 | 34.9833 | C4 grass, tallgrass prairie | Xu et al., 2012 |
| Jornada | -106.85 | 32.6 | SubTropical semi-desert steppe C4 grass | Hui and Jackson, 2005 |
| Montecillo | -98.91 | 19.46 | Forest–meadow–paramo C4 grass | Hui and Jackson, 2005 |
| Duolun | 116.283 | 42.0333 | Semi-arid C3 grass | Xu et al., 2016 |
| Matador | -102.72 | 50.7 | Temperate dry steppe C3 grass | Hui and Jackson, 2005 |
| Tumugi | 123 | 46.1 | Cold desert steppe C3 grass | Hui and Jackson, 2005 |

**Table S2**. Magnitudes of the carbon flux, carbon allocation ratios, and leaf area index (LAI) obtained by CABLE using the resource limitation carbon allocation model (RES) and fixed coefficient model (FIX). Differences between variables are shown in brackets. NEP: net ecosystem productivity; GPP: gross primary production, Ra: autotrophic respiration; NPP: net primary production; Rh: heterotrophic respiration; ER: ecosystem respiration, ER=Ra+Rh; aleaf: carbon allocation to leaves; awood: carbon allocation to wood; afroot: carbon allocation to roots; ENF: evergreen needleleaf forest; EBF: evergreen broadleaf forest; DNF: deciduous needleleaf forest; DBF: deciduous broadleaf forest; SHB: shrub; C3: C3 grass; C4: C4 grass; TDR: tundra; CROP: C3 crop.

| PFT | NEP (Pg C yr^–1^) | |  | GPP (Pg C yr^–1^) | |  | Ra (Pg C yr^–1^) | |  | NPP (Pg C yr^–1^) | |  | Rh (Pg C yr^–1^) | |
| --- | --- | --- | --- | --- | --- | --- | --- | --- | --- | --- | --- | --- | --- | --- |
|  | FIX | RES (Diff) |  | FIX | RES (Diff) |  | FIX | RES (Diff) |  | FIX | RES (Diff) |  | FIX | RES (Diff) |
| ENF | 0.751 | 0.594(–0.157**) |  | 26.490 | 32.360(+5.870**) |  | 15.786 | 18.335(+2.549**) |  | 10.704 | 14.025(+3.321**) |  | 9.953 | 13.431(+3.478**) |
| EBF | 0.425 | 0.867(+0.442*) |  | 48.205 | 53.438(+5.233**) |  | 34.497 | 39.350(+4.852**) |  | 13.708 | 14.089(+0.381**) |  | 13.283 | 13.222(–0.061) |
| DNF | 0.009 | 0.016(+0.006) |  | 0.514 | 0.553(+0.039**) |  | 0.233 | 0.245(+0.012**) |  | 0.281 | 0.309(+0.027**) |  | 0.272 | 0.293(+0.021**) |
| DBF | 0.232 | 0.342(+0.110**) |  | 8.951 | 9.585(+0.634**) |  | 4.816 | 5.078(+0.262**) |  | 4.135 | 4.507(+0.372**) |  | 3.903 | 4.165(+0.262**) |
| SHB | 0.343 | 0.309(–0.035) |  | 13.047 | 10.318(–2.729**) |  | 7.900 | 6.274(–1.626**) |  | 5.147 | 4.044(–1.103**) |  | 4.804 | 3.735(–1.069**) |
| C3 | 0.200 | 0.174(–0.026) |  | 16.653 | 18.147(+1.494**) |  | 8.510 | 9.615(+1.106**) |  | 8.143 | 8.531(+0.388**) |  | 7.943 | 8.357(+0.414**) |
| C4 | 0.107 | 0.113(+0.006) |  | 8.771 | 15.219(+6.448**) |  | 4.707 | 8.237(+3.530**) |  | 4.065 | 6.982(+2.197**) |  | 3.957 | 6.869(+2.912**) |
| TDR | 0.055 | 0.111(+0.056**) |  | 6.485 | 5.064(–1.421**) |  | 2.895 | 2.101(–0.794**) |  | 3.590 | 2.963(–0.627**) |  | 3.534 | 2.852(–0.683**) |
| CROP | 0.357 | 0.352 (–0.005) |  | 10.157 | 15.691(+5.534**) |  | 5.130 | 8.228(+3.098**) |  | 5.027 | 7.464(+2.436**) |  | 4.671 | 7.112(+2.441**) |
| Global | 2.480 | 2.876(+0.397) |  | 139.273 | 160.375(+21.102**) |  | 84.473 | 97.461(+12.989**) |  | 54.801 | 62.913(+8.112**) |  | 52.322 | 60.036(+7.715**) |

| PFT | ER (Pg C yr^–1^) | |  | aleaf (–) | |  | awood (–) | |  | afroot (–) | |  | LAI (m^2^/m^2^) | |
| --- | --- | --- | --- | --- | --- | --- | --- | --- | --- | --- | --- | --- | --- | --- |
|  | FIX | RES (Diff) |  | FIX | RES (Diff) |  | FIX | RES (Diff) |  | FIX | RES (Diff) |  | FIX | RES (Diff) |
| ENF | 25.739 | 31.766(+6.027**) |  | 0.250 | 0.326(+0.077**) |  | 0.400 | 0.486(+0.086**) |  | 0.350 | 0.187(–0.163**) |  | 3.073 | 5.145(+2.072**) |
| EBF | 47.78 | 52.572(+4.792**) |  | 0.198 | 0.248(+0.049**) |  | 0.351 | 0.658(+0.307**) |  | 0.451 | 0.094(–0.357**) |  | 3.808 | 4.697(+0.889**) |
| DNF | 0.505 | 0.538(+0.033**) |  | 0.254 | 0.251(–0.003) |  | 0.353 | 0.177(–0.176**) |  | 0.393 | 0.572(+0.179**) |  | 1.396 | 1.371(–0.025) |
| DBF | 8.719 | 9.243(+0.524**) |  | 0.173 | 0.177(+0.004*) |  | 0.310 | 0.407(+0.097**) |  | 0.517 | 0.416(–0.101**) |  | 1.662 | 1.792(+0.130**) |
| SHB | 12.704 | 10.009(–2.695**) |  | 0.114 | 0.098(–0.016**) |  | 0.335 | 0.121(–0.214**) |  | 0.551 | 0.781(+0.229**) |  | 0.869 | 0.625(–0.244**) |
| C3 | 16.453 | 17.972(+1.519**) |  | 0.149 | 0.151(+0.002**) |  | - | - |  | 0.851 | 0.849(–0.002) |  | 1.287 | 1.382(+0.095**) |
| C4 | 8.664 | 15.106(+6.442**) |  | 0.147 | 0.172(+0.025**) |  | - | - |  | 0.853 | 0.828(–0.025**) |  | 0.772 | 1.394(+0.621**) |
| TDR | 6.429 | 4.953(–1.476**) |  | 0.304 | 0.234(–0.070**) |  | 0.135 | 0.160(+0.025**) |  | 0.561 | 0.606(+0.045**) |  | 1.334 | 0.897(–0.437**) |
| CROP | 9.801 | 15.34(+5.539**) |  | 0.218 | 0.255(+0.037**) |  | - | - |  | 0.782 | 0.745(–0.037**) |  | 1.049 | 1.769(+0.720**) |
| Global | 136.795 | 157.497(+20.702**) |  | 0.195 | 0.210(+0.015**) |  | 0.193 | 0.220(+0.027**) |  | 0.611 | 0.570(–0.042**) |  | 1.733 | 2.225(+0.492**) |

**Highly significant change (*p* < 0.01); *Significant change (*p* < 0.05)

**Table S3**. Trends in the carbon flux, carbon allocation ratios, and leaf area index (LAI) obtained by CABLE using the resource limitation carbon allocation model (RES) and fixed coefficient model (FIX). Differences between variables are shown in brackets. NEP net ecosystem productivity; GPP: gross primary production, Ra: autotrophic respiration; NPP: net primary production; Rh: heterotrophic respiration; ER: ecosystem respiration, ER=Ra+Rh; aleaf: carbon allocation to leaves; awood: carbon allocation to wood; afroot: carbon allocation to roots; ENF: evergreen needleleaf forest; EBF: evergreen broadleaf forest; DNF: deciduous needleleaf forest; DBF: deciduous broadleaf forest; SHB: shrub; C3: C3 grass; C4: C4 grass; TDR: tundra; CROP: C3 crop.

| PFT | NEP Trend (Pg C yr^–2^) | |  | GPP Trend (Pg C yr^–2^) | |  | Ra Trend (Pg C yr^–2^) | |  | NPP Trend (Pg C yr^–2^) | |  | Rh Trend (Pg C yr^–2^) | |
| --- | --- | --- | --- | --- | --- | --- | --- | --- | --- | --- | --- | --- | --- | --- |
|  | FIX | RES (Diff) |  | FIX | RES (Diff) |  | FIX | RES (Diff) |  | FIX | RES (Diff) |  | FIX | RES (Diff) |
| ENF | –0.008 ^*^ | 0.000(+0.008) |  | 0.065^**^ | 0.062^**^ (–0.002) |  | 0.053^**^ | 0.046^**^(–0.006) |  | 0.012^**^ | 0.016^**^(+0.004) |  | 0.020^**^ | 0.016^**^(–0.004) |
| EBF | –0.043^**^ | –0.037^**^ (+0.006) |  | –0.018 | 0.003(+0.021) |  | 0.010 | 0.021^*^(+0.012) |  | –0.028^**^ | –0.018(+0.009) |  | 0.015^**^ | 0.019^**^(+0.004) |
| DNF | 0.001^*^ | 0.000(–0.0005) |  | 0.003^**^ | 0.002^**^(–0.0004) |  | 0.001^**^ | 0.001^**^ (0.000) |  | 0.001^**^ | 0.001(+0.000) |  | 0.001^**^ | 0.001^**^ (0.000) |
| DBF | –0.005 | –0.004(+0.001) |  | 0.022^**^ | 0.024^**^ (+0.002) |  | 0.016^**^ | 0.016^**^(+0.001) |  | 0.006^*^ | 0.008^**^(+0.002) |  | 0.011^**^ | 0.012^**^(+0.001) |
| SHB | 0.009^**^ | 0.012^**^ (+0.003) |  | 0.073^**^ | 0.079^**^ (+0.006) |  | 0.049^**^ | 0.053^**^(+0.004) |  | 0.023^**^ | 0.026^**^(+0.003) |  | 0.015^**^ | 0.014^**^(–0.001) |
| C3 | –0.006 | –0.007(–0.0018) |  | 0.053^**^ | 0.048^**^ (–0.005) |  | 0.034^**^ | 0.032^**^(–0.002) |  | 0.019^**^ | 0.016^**^(–0.003) |  | 0.024^**^ | 0.023^**^(–0.001) |
| C4 | 0.001 | 0.005(+0.004) |  | 0.036^*^ | 0.054^**^ (+0.018) |  | 0.021^**^ | 0.032^**^ (+0.011) |  | 0.015 | 0.022^*^(+0.008) |  | 0.014^**^ | 0.017^**^(+0.003) |
| TDR | 0.002^**^ | 0.004^**^ (+0.001) |  | 0.026^**^ | 0.027^**^ (+0.001) |  | 0.015^**^ | 0.014^**^(–0.001) |  | 0.011^**^ | 0.013^**^(+0.002) |  | 0.009^**^ | 0.009^**^(+0.001) |
| CROP | –0.010 | –0.012^*^(–0.0016) |  | 0.043^**^ | 0.054^**^ (+0.011) |  | 0.026^**^ | 0.036^**^ (+0.010) |  | 0.017^**^ | 0.018^**^(+0.001) |  | 0.027^**^ | 0.030^**^(+0.003) |
| Global | –0.058^**^ | –0.038(+0.020) |  | 0.303^**^ | 0.355^**^ (+0.052) |  | 0.225^**^ | 0.252^**^ (+0.027) |  | 0.078^**^ | 0.103^**^(+0.025) |  | 0.136^**^ | 0.141^**^(+0.005) |

| PFT | ER Trend (Pg C yr^–2^) | |  | aleaf Trend (100yr^–1^) | |  | awood Trend (100yr^–1^) | |  | afroot Trend (100yr^–1^) | |  | LAI | |
| --- | --- | --- | --- | --- | --- | --- | --- | --- | --- | --- | --- | --- | --- | --- |
|  | FIX | RES (Diff) |  | FIX | RES (Diff) |  | FIX | RES (Diff) |  | FIX | RES (Diff) |  | FIX | RES (Diff) |
| ENF | 0.073** | 0.062**(–0.011) |  | 0.000 | –0.028**(–0.028) |  | 0.000 | 0.013(+0.013) |  | 0.000 | 0.015(+0.015) |  | 0.004** | 0.003** (–0.001) |
| EBF | 0.025* | 0.04** (+0.015) |  | 0.000 | 0.022**(+0.022) |  | 0.000 | 0.000(0.000) |  | 0.000 | –0.022(–0.022) |  | -0.005** | –0.005** (0.000) |
| DNF | 0.002** | 0.002** (0.000) |  | –0.021 | –0.016(+0.005) |  | 0.007** | –0.008(–0.015) |  | 0.014 | 0.024(+0.010) |  | 0.004** | 0.003*(–0.001) |
| DBF | 0.027** | 0.028**(+0.001) |  | 0.054** | 0.051**(–0.003) |  | -0.020** | 0.030*(+0.050) |  | 0.033** | –0.080**(–0.047) |  | 0.002** | 0.002** (0.000) |
| SHB | 0.064** | 0.067**(+0.003) |  | 0.033** | 0.050**(+0.017) |  | -0.011** | 0.076**(+0.087) |  | -0.020** | –0.124**(–0.104) |  | 0.004** | 0.005**(+0.001) |
| C3 | 0.058** | 0.055**(–0.003) |  | 0.013** | –0.007*(–0.020) |  | - | - |  | –0.013 | 0.007**(+0.020) |  | 0.002** | 0.001** (–0.001) |
| C4 | 0.035** | 0.049**(+0.014) |  | 0.045** | 0.083**(+0.038) |  | - | - |  | –0.045* | –0.083(–0.038) |  | 0.003 | 0.005**(+0.002) |
| TDR | 0.024** | 0.023**(–0.001) |  | –0.033** | 0.006(+0.039) |  | 0.012 | 0.052**(+0.040) |  | 0.027* | –0.052**(–0.079) |  | 0.003** | 0.004**(+0.001) |
| CROP | 0.053** | 0.066**(+0.013) |  | –0.022** | –0.014(+0.008) |  | - | - |  | 0.022** | 0.014(–0.008) |  | 0.003** | 0.003**(–0.0006) |
| Global | 0.361** | 0.393**(+0.032) |  | 0.008** | 0.015**(+0.007) |  | –0.001 | 0.020**(+0.020) |  | –0.007** | –0.034**(–0.027) |  | 0.002** | 0.002**(+0.0002) |

**Highly significant change (*p* < 0.01); *Significant change (*p* < 0.05)

**References**

Beer, C., et al. Terrestrial gross carbon dioxide uptake: Global distribution and covariation with climate, Science, 329, 834–838 (2010).

da Costa, A. C. et al. Ecosystem respiration and net primary productivity after 8–10 years of experimental through-fall reduction in an eastern Amazon forest. Plant Ecology & Diversity 7, 7-24 (2014).

De Kauwe, M. G. et al. Where does the carbon go? A model–data intercomparison of vegetation carbon allocation and turnover processes at two temperate forest free‐air CO2 enrichment sites. New Phytologist 203, 883-899 (2014).

Doughty, C. E. et al. Drought impact on forest carbon dynamics and fluxes in Amazonia. Nature 519, 78-82 (2015).

Jung, M., Reichstein, M., Margolis, H. A., Cescatti, A., Richardson, A. D., Arain, M. A., ... & Gianelle, D. Global patterns of land‐atmosphere fluxes of carbon dioxide, latent heat, and sensible heat derived from eddy covariance, satellite, and meteorological observations. Journal of Geophysical Research: Biogeosciences, 116(G3) (2011)..

Jung M, Reichstein M, Schwalm C R, Huntingford C, Sitch S, Ahlström A, Arneth A, Camps-Valls G, Ciais P and Friedlingstein P Compensatory water effects link yearly global land CO2 sink changes to temperature Nature 541 516-20 (2017).

Le Quéré C, Raupach M R, Canadell J G, Marland G, Bopp L, Ciais P, Conway T J, Doney S C, Feely R A and Foster P Trends in the sources and sinks of carbon dioxide Nature Geoscience 2 831-6 (2009).

Hui, D. and Jackson, R. B. Geographical and interannual variability in biomass partitioning in grassland ecosystems: a synthesis of field data. New Phytologist 169, 85-93 (2006).

Sitch S, Huntingford C, Gedney N, Levy P, Lomas M, Piao S, Betts R, Ciais P, Cox P and Friedlingstein P Evaluation of the terrestrial carbon cycle, future plant geography and climate‐carbon cycle feedbacks using five Dynamic Global Vegetation Models (DGVMs) Global Change Biology 14 2015-39 (2008).

Xu, X. et al. Interannual variability in responses of belowground NPP and NPP partitioning to long-term warming and clipping in a tallgrass prairie. Global Change Biol 18, 1648-1656 (2012).

Xu, Z. et al. Experimentally increased water and nitrogen affect root production and vertical allocation of an old-field grassland. Plant and Soil, 1-12 (2016).
